# Production and Purification of Artificial Circular RNA Sponges for Application in Molecular Biology and Medicine

**DOI:** 10.3390/mps3020042

**Published:** 2020-05-26

**Authors:** Janina Breuer, Oliver Rossbach

**Affiliations:** Institute of Biochemistry, Faculty of Biology and Chemistry, University of Giessen, Heinrich-Buff-Ring 17, D-35392 Giessen, Germany; janina.breuer@chemie.bio.uni-giessen.de

**Keywords:** circular RNA, circular RNA sponges, microRNA, RNA therapy, molecular sponge, circRNA production, artificial circular RNA sponge, artificial circRNA, ciRS

## Abstract

Characterized by their covalently closed structure and thus an elevated stability compared to linear RNA molecules, circular RNAs (circRNAs) form a novel class of mainly non-coding RNAs. Although the biological functions of naturally occurring circRNAs are largely unknown, they were reported to act as molecular sponges, sequestering microRNAs (miRNAs), resulting in a de-repression of target mRNAs. Taking these characteristics of naturally occurring circRNAs into account, artificial circRNAs could be a potential tool in molecular biology and medicine. Using the Hepatitis C virus (HCV) as a model system, this application of artificial circular RNAs was demonstrated. The virus requires cellular miRNA miR-122 for its life cycle, and circRNAs specifically engineered to efficiently sequester this miRNA impacted viral propagation. Since in this context the production of engineered circRNA remains the limiting factor, we present a method to produce and efficiently purify artificial circRNA sponges (ciRS) *in vitro*. In this protocol we provide insights into a small-scale and large-scale production technique of artificial circular RNA sponges relying on *in vitro* transcription and RNA ligation.

## 1. Introduction

Circular RNAs (circRNAs) form a novel class of mainly non-coding RNAs. Naturally occurring circRNAs arise by an alternative splicing mechanism termed “backsplicing” [1] or “head-to-tail-splicing” [2]. A splice donor is covalently joined to an upstream instead of a downstream splice acceptor site relying on canonical splicing signals [1,2,3,4,5]. Therefore, a circularized RNA molecule is created, which has neither a 5’ cap nor a 3’ poly(A) tail, but forms a covalently closed structure. This conformation appears to make circRNAs resistant to exonucleolytic degradation, resulting in an elevated stability compared to linear RNAs [1,6].

The first evidence of the existence of circRNAs was reported in 1976, demonstrating plant viroid genomes are composed of single-stranded covalently closed RNA molecules [7]. After their discovery, circRNAs were underestimated as events of aberrant splicing with neglectable biological relevance, and only few candidates of naturally occurring circular RNA, such as SRY, ETS-1, and DCC, were found [6,8,9,10]. Since 2012, advances in RNA sequencing (RNA-seq) methodologies and bioinformatics have gained novel insights into the circRNA world by identifying thousands of human exonic circRNAs [1,11,12]. To not only identify, but rather increase the functional understanding of circRNAs, a broad range of circRNA expression systems was established. Relying on specifically designed overexpression vectors, desired circRNAs can be produced in cell culture (hereafter termed “*in vivo*”) by both a spliceosome-dependent exon circularization strategy [2,13,14] and a spliceosome-independent strategy based on engineered ribozymes derived from the tRNA splicing machinery (e.g., the “tornado” system) [15]. Additionally, strategies relying on circRNA production via cell-free systems (hereafter termed “*in vitro*”) using recombinant phage RNA polymerase-mediated transcription and circularization by either employing genetically engineered autocatalytic group I introns (e.g., the permutated exon-intron (PEI) system) [14,16,17,18] or enzymatic intramolecular transcript ligation using recombinant RNA ligases [14,19] gained importance to further characterize circRNA utilities.

Although the biological functions of endogenous circRNAs are still largely unknown, there are several lines of evidence showing that circRNAs have diverse important regulatory and developmental roles [2,12,20,21,22]. In this context, the cellular circRNA CDR1as/ciRS-7 was identified as a molecular sponge or decoy sequestering the cytoplasmic miRNA miR-7 via 70 highly conserved binding sites [2,13]. The observed co-expression of CDR1as/ciRS-7 with miR-7 in neocortical and hippocampal neurons leads to a suppression of miR-7 functions, thereby de-repressing natural miR-7 targets. CDR1as/ciRS-7 knockout mice display misregulation of other miRNAs, dysfunctional synaptic transmission, and sensimotor gating, which has also been associated with human neuropsychiatric disorders. These results indicate the importance of CDR1as/miR-7-interactions for normal brain functions [23]. Furthermore, circRNAs have been shown to act as sponges for RNA-binding proteins (RBPs), as well as nuclear transcriptional regulators—illustrating their relevance in the context of the regulatory networks governing gene expression [22,24,25].

Considering these functions and characteristics of naturally occurring circRNAs, artificial circRNAs could be a potential tool for molecular biology and medicine applied for the development of new strategies to combat human diseases such as viral infections or cancer. In this context, our laboratory utilized the Hepatitis C virus (HCV) as a model system in a proof of principle study. The single-stranded RNA genome of the hepatocyte-specific virus is bound by miR-122 at two distinct binding sites at its 5’-end (besides others), thereby protecting the viral RNA from exonucleolytic degradation and enhancing viral translation. Sequestration of miR-122 had already been shown to inhibit viral propagation in patients using the first locked nucleic acid (LNA)-containing antisense miRNA drug (Miravirsen). As a novel complementary approach, artificial circular RNA sponges (ciRS) produced as described in this protocol, were shown to efficiently bind and sequester the cellular miR-122 *in vitro* and *in vivo*. When transfected into cells, these circRNAs are both present in the nucleus and the cytoplasm, and are more stable than their linear counterparts [19].

With the aim of targeting miRNAs misregulated within human diseases, artificial circular RNA sponges represent an efficiently functioning and inexpensive alternative to commercially available LNA-containing anti-miRNA drugs. Furthermore, circular RNA sponges facilitate functional analyses of cellular miRNAs by sequestration comparable to siRNA-based approaches to decrease the target mRNA level. However, since the production of engineered circRNA remains the limiting factor, this protocol provides insights into an efficient production technique of artificial circular RNA sponges relying on *in vitro* transcription and RNA ligation suitable for small and middle-sized RNAs. In this article, a small-scale as well as a large-scale production procedure will be described, typically yielding up to 20 µg and 350 µg of artificial circular RNA sponges, respectively.

## 2. Experimental Design

### 2.1. Materials

#### 2.1.1. Kits and Enzymes

- HiScribe T7 High Yield RNA Synthesis Kit (New England Biolabs; Ipswich, MA, USA; Cat. no.: E2040S)
- Ribonuclease R (Lucigen Corporation, Middleton, WI, USA; Cat. no.: RNR07250)
- RNaseOUT Recombinant Ribonuclease Inhibitor (Thermo Fisher Scientific, Waltham, MA, USA; Cat. no.: 10777019)
- RQ1 RNase-free DNase (Promega Corporation; Madison, WI, USA; Cat. no.: M6101)
- T4 RNA Ligase (Thermo Fisher Scientific, Waltham, MA, USA; Cat. no.: EL0021)

#### 2.1.2. Reagents

- Adenosine triphosphate (ATP; Roche Diagnostics GmbH, Basel, Switzerland; Cat. no. 11140965001)
- Dithiothreitol (DTT; 100 mM; Thermo Fisher Scientific, Waltham, MA, USA; Cat. no.: R0862)
- Ethidium bromide solution 1% (Carl Roth GmbH + Co. KG, Karlsruhe, Germany; Cat. no.: 2218.1)
- Guanosine 5′-monophosphate disodium salt hydrate (GMP; Merck KGaA; Darmstadt, Germany; Cat. no.: G8377)
- Lipofectamine 2000 Transfection Reagent (Thermo Fisher Scientific, Waltham, MA, USA; Cat. no.: 11668027)
- Polyethylenimine (Merck KGaA; Darmstadt, Germany; Cat. no.: 764604-1G)
- SYBR Gold Nucleic Acid Gel Stain (Thermo Fisher Scientific, Waltham, MA, USA; Cat. no.: S11494)

#### 2.1.3. Buffers

- Urea solution (50% urea (*w*/*v*) in 1 × Tris-Borate-EDTA (TBE))
- 20% denaturing polyacrylamide solution (20% polyacrylamide (19:1 acrylamide/bisacrylamide) in 1× TBE containing 50% urea (*w*/*v*))
- 10 × circRNA annealing buffer (100 mM Tris pH 7.5, 500 mM NaCl)
- Formamide loading buffer (1 × TBE, 90% formamide, 0.05% bromphenol blue (*w*/*v*), 0.05% xylene cyanol (*w*/*v*))
- PK buffer (100 mM Tris/HCl pH 7.5, 150 mM NaCl, 12.5 mM EDTA, 1% SDS)

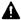
 Ensure used materials are RNase-free prior to use.

### 2.2. Equipment

- Bunsen burner
- Corning Costar Spin-X Plastic Centrifuge Tube Filters (Merck KGaA; Darmstadt, Germany Cat. no.: CLS8160)
- Eppendorf Tubes 5.0 mL Snap cap (Eppendorf AG, Hamburg, Germany; Cat. no.: 0030119401)
- mini Quick Spin RNA Columns (Merck KGaA; Darmstadt, Germany Cat. no.: 11814427001)
- Phase Lock Gel Heavy 2 mL Tubes (QuantaBio; Beverly, MA, USA; Cat. no.: 2302830)
- Pre-coated TLC plates CEL 400-10 UV254 (Macherey Nagel GmbH & Co. KG; Düren, Deutschland; Cat. no.: 808083)
- Sterican Gr. 18, G 26 × 1""/ø 0,45 × 25 mm (B. Braun Melsungen AG, Melsungen, Germany; Cat. no.: 4657683)

## 3. Procedure

### 3.1. Engineering of Templates for Artificial Circular RNA Sponges

**Figure 1:**
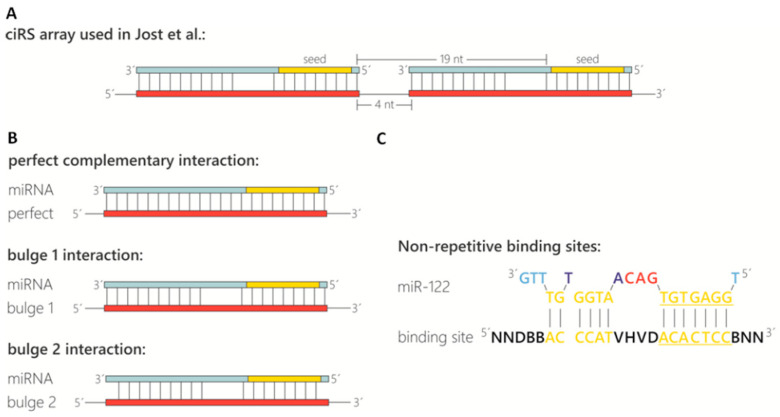
Design of miRNA binding sites contained in circular RNA sponges. (**A**) Representation of the ciRS array used by Jost et al. [19] for sequestration of miR-122 (light blue). The array is characterized by miRNA binding sites (red) containing a bulge between nucleotides (nt) 10–12 and a spacing of 4 nt between binding sites resulting in a distance of 19 nt between the adjacent miRNA seed sequences (yellow). (**B**) Schematic representation of different potential miRNA binding sites with distinct complementarity to the target miRNA. (**C**) Non-repetitive miR-122 binding site for more efficient circularization.

**Figure 2:**
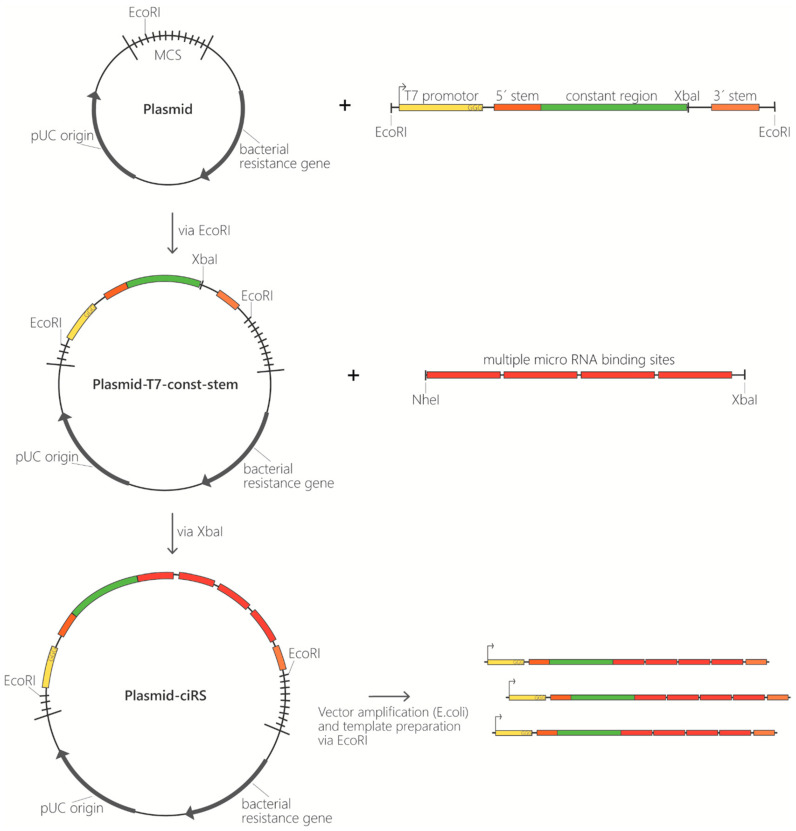
Template generation for ciRS production. Overview of cloning procedure for ciRS construction starting with insertion of the T7 promotor (yellow), a double-stranded stem-loop region (5′ stem and 3′ stem; orange), and a constant region (green) via *Eco*RI into a multipurpose vector backbone. Afterwards *Xba*I is used to insert the designed ciRS array consisting of multiple miRNA binding sites for the miRNA of interest. The final ciRS-vector is amplified, and templates are prepared.

**Figure 3:**
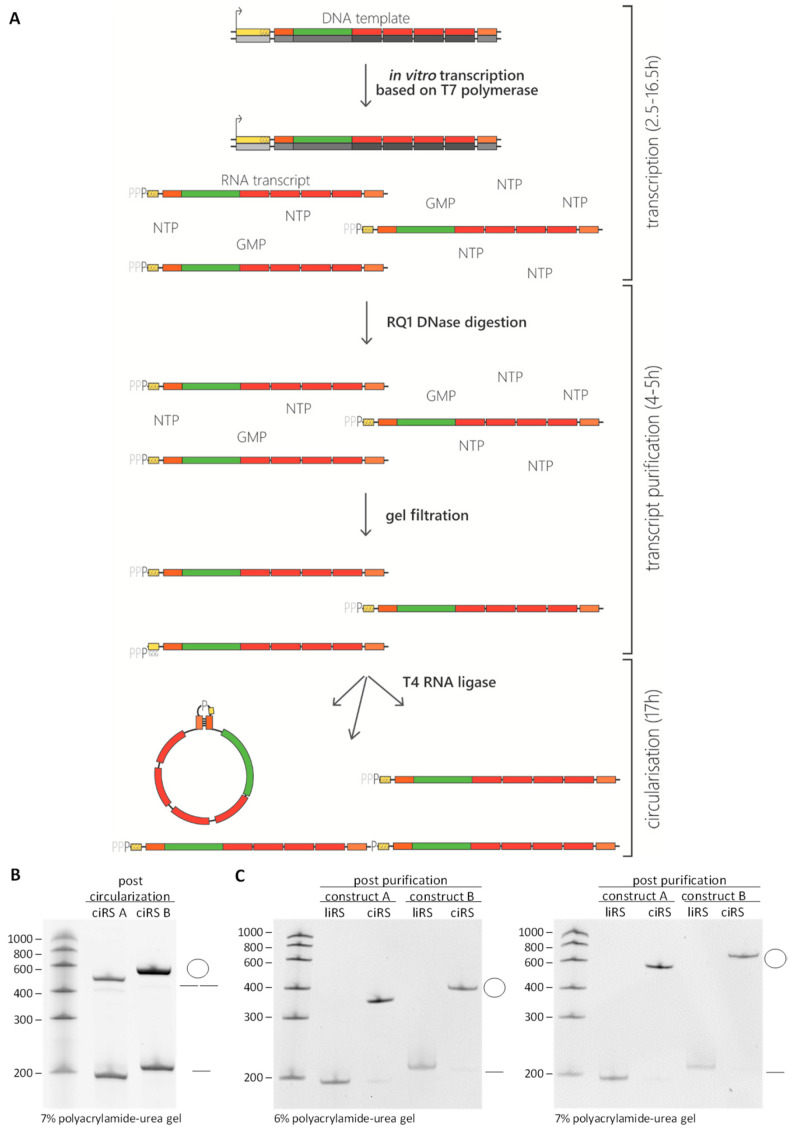
*In vitro* preparation of circular RNA sponges. (**A**) Schematic overview of the ciRS production procedure—starting with *in vitro* transcription with 10-fold excess of GMP, followed by DNase digestion to remove template DNA, gel filtration to clear away excess nucleotides (NTP, GMP) derived from the transcription reaction, and ligation of the purified transcript. Considering the activity of the T4 RNA ligase, transcript ligation can result in both linear monomers and dimers of the transcript and circularized transcripts (indicated by the dash/double dash or the circle in (B) and (C)). (**B**) *In vitro* circularisation reaction of two exemplary constructs was analysed by 7% polyacrylamide-urea gel electrophoresis, followed by ethidium bromide staining showing linear and circular transcripts with a circularisation efficiency of ~ 40%–60% and only small amounts of linear dimers detectable. As described in Section 3.1, the transcripts are characterized by a constant region and four miRNA binding sites but differ in the presence of a double-stranded stem-loop region increasing the circularisation efficiency (construct A: 197 nt, lacking the 11 nt 3′stem-loop-sequence; construct B: 208 nt, with stem-loop). (**C**) Circular and linear isoforms of the RNA sponges (ciRS and liRS) were purified, and quality was verified on analytic 6% and 7% polyacrylamide-urea gels by ethidium bromide staining. While the mobility of linear RNAs remains unchanged compared to the RNA ladder, the mobility of circular RNA appears lower in higher percentage polyacrylamide-urea gels, resulting in a shift of the circular RNA when comparing the polyacrylamide-urea gels with different concentrations.

1. *Design of microRNA binding sites*. The initial design of the miRNA binding sites present in the circular RNA sponges, or the corresponding control sequences, is crucial to the procedure. Therefore, several features and limitations have to be considered. The goal is to design an array of miRNA binding sites that is specific for binding to the miRNA of interest, has sufficient thermodynamic stability to be able to compete with the endogenous miRNA targets, and is able to harbour the maximum number of binding sites without impairing *in vitro* circularization.
  - *Number of miRNA binding sites:* The natural prototype of a circular miRNA sponge, ciRS-7/CDR1as, has ~70 miR-7 binding sites [2,13]. This large number cannot be recapitulated in artificial circRNAs. The main reasons are on one hand, the transcript size impairing the circularisation efficiency and on the other hand, the repetitive nature of these arrays interfering with most procedures in molecular biology, such as cloning, PCR or Sanger sequencing. Therefore, we found that an array of 4 to 8 miRNA binding sites is optimal for the production of artificial circular RNA sponges. Furthermore, as eight binding sites impaired efficient circularization, four binding sites were used in this protocol. Despite sharing the same basic structure as used by Jost et al. 2018, this protocol is based on the production of two exemplary ciRS (construct A and construct B) containing four binding sites for an miRNA of interest and differing in the presence of a double-stranded stem-loop sequence (see Section 3.1, paragraph 2.) [19] Another benefit is that a sequence stretch containing four typical miRNA binding sites (including spacing and flanking restriction sites) can be easily ordered via DNA oligonucleotide synthesis. These 4-binding site arrays can subsequently be multimerized by cloning or *in vitro* ligation. Generally, shorter transcripts tend to circularize more efficiently compared to longer RNAs.
  - *Binding site spacing:* Besides the sequence composition and thus degree of complementarity to the target miRNA, the optimal spacing of miRNA binding sites is considered here. Based on analyses of natural miRNA:mRNA interactions (16–20 nt between seed sequences; [26]), a spacing of 4 nt between binding sites was chosen, resulting in a distance of 19 nt between the adjacent miRNA seed sequences (Figure 1A).
  - *Binding site types and specificity:* The configuration of the miRNA binding site itself is also an important feature (Figure 1B). In sequestration of miR-122 from HCV, a bulged binding site lacking complementarity at nucleotides 10–12 was most effective [19]. Although a perfectly complementary site might appear thermodynamically superior, the occurrence of miRNAs in a complex with Ago2 in the cytoplasm renders bulged binding sites most effective [19,27]. In addition, a perfectly complementary binding site is assumed to be cleaved by the miRNA-RISC complex [27,28], although we did not observe cleavage of the perfectly complementary binding sites in any circular RNA sponges in cells [19]. Another feature to consider is the complementarity at the miRNA 5’ and 3’ ends (Figure 1B, compare “bulged 1” and “bulged 2”). Base-paired ends of binding sites were reported to cause tailing or trimming of the targeted miRNA, which can lead to miRNA degradation [29,30]. In the context of the miRNA sponges tested, we never observed tailing, trimming or degradation of miR-122 [19]. To test the specificity of the chosen miRNA binding sites and to prevent off-target effects, a miRNA binding site can also be designed and/or tested using a publicly available bioinformatics tool tailored to generate and test artificial miRNA binding sites. The “miRNAsong” tool (MicroRNA SpONge Generator and tester) is a valuable resource in this regard [31].
  - *Binding site repetitiveness for longer miRNA binding site arrays* (**OPTIONAL STEP**)*:* In case longer stretches of miRNA binding site arrays are desired, the perfectly repetitive units of the binding sites and spacer sequences (described above; Figure 1A,B) are not feasible. A very effective approach to prevent repetitiveness and to allow gene synthesis of longer fragments is randomization of bases in the binding site that are not essential for the interaction with the miRNA. Based on biophysical data obtained from the analysis of cellular miRNA:mRNA interactions [32], we determined the bases within miR-122 that are supposedly not essential for an efficient interaction and randomized them across an array of up to 20 binding sites. Including G:U wobble base-pairs in the non-seed interaction region may help to achieve a degree of sequence diversity for gene synthesis approaches. Circularization efficiency was much higher with these randomized arrays compared to a similar number of repetitive binding site arrays. This approach might be useful for miRNA binding site arrays that compete with weaker endogenous sites, e.g., on cellular mRNAs, or when aiming to shift binding equilibria in the cell.
  - *Negative controls:* The last important consideration, when designing circRNA sponges to sequester miRNAs, is the appropriate negative controls. Besides efforts to keep the length and overall base composition of the control sponges as similar as possible compared to the actual miRNA sponge, there are different concepts of control design. In Jost et al 2018, we used the actual binding site array and randomized the complete sequence stretch using publicly available sequence randomizers. To ensure no miR-122 binding sites were randomly generated, a sequence alignment of the miRNA on the randomized sequences was performed. Another approach that generates control binding sites with drastically reduced miRNA affinity is targeted mutation of the complete seed sequence (nt 2–8 of the miRNA). Note that seed mutant binding sites retain a certain non-canonical miRNA-binding activity [19]. Optimally, several different negative controls should be tested in an experimental set, since unwanted secondary RNA structures generated by chance may impair the *in vitro* circularization efficiency.
2. *Template generation as starting point for ciRS production in vitro.* Conventionally, templates contain the following elements: a T7 promotor sequence for *in vitro* transcription, several binding sites complementary to the miRNA of interest, and optionally a double-stranded stem-loop sequence at the respective 5′- and 3′-termini to enhance circularization efficiency (Figure 2). The latter is characterized by a double-stranded stem of 11 nt with a non-basepaired overhang of 5 nt on both ends forming the open loop of 10 nt in total (contained in construct B, Figure 3). Despite using a double-stranded stem-loop, other circularization strategies (e.g., using splint oligonucleotides that anneal at both ends) can be used. Note that depending on, e.g., the length and sequence of individual templates, circularisation can also be observed for transcripts lacking the double-stranded stem-loop and without using a splint oligonucleotide (see Figure 3, construct A). Despite the mentioned features, we also typically include a region that is universal for all miRNA-sponge constructs and negative controls of a certain set (“constant region”), which serves as a binding site for PCR primers, antisense probes in northern blotting or *in situ* hybridization procedures. This region spans 63 nt in the templates used here. Moreover, it enables a reliable comparative quantification of different constructs. Adding flanking restriction sites enables cloning into a plasmid of choice. The described sequence elements can be obtained from any company offering gene synthesis services and are available within a multipurpose vector backbone upon request. Plasmids can be purified at a large scale, and the *in vitro* transcription template can be excised and gel-purified via agarose gel electrophoresis (Figure 2). Afterwards, templates are subjected to *in vitro* transcription reaction.

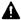
 CRITICAL STEP: Unless described otherwise, all of the following procedures should be performed on ice!

### 3.2. In Vitro Transcription. Time for Completion: 2.5–16.5 h

*In vitro* transcription is performed using the HiScribe T7 High Yield RNA Synthesis Kit and 10-fold excess of guanosine 5′-monophosphate (GMP). The GMP-initiated *in vitro* transcription aims to obtain mainly 5′-monophosphorylated linear RNA transcripts which can be directly used for RNA circularization. Since all T7 polymerase-created *in vitro* transcripts start with three guanosines, a stochastic subset of RNAs will be initiated with GMP instead of guanosintriphosphate (GTP) (Figure 3A). Thereby, the two enzymatic steps of de- and rephosphorylation used in other protocols are circumvented [14,33,34]. Assuming the active centre of the RNA-polymerase accepts GMP and GTP with the same affinity, transcription with 10-fold excess of GMP results in a mixture of total transcripts consisting of 90% monophosphate 5′ends. The latter can be subjected to circularization reaction using the T4 RNA ligase. Note that GMP cannot be used for the polymerization itself. The transcription reaction contains the following components: 1/10 vol. 10 × T7 Reaction Buffer, 10 mM final concentration of ATP, GTP, CTP, and UTP (100 mM), 100 mM final concentration of GMP (400 mM), final concentration of 130 nM (**small scale**: 200 ng; **large scale**: 1 µg) of DNA template, and 1/10 vol. of T7 RNA Polymerase Mix (10×). Adjust to final volume with RNase-free water:

- **small scale**: prepare 20 µL total volume of the transcription reaction
- **large scale:** prepare 100 µL total volume of the transcription reaction

Incubate 2 h up to overnight at 37 °C.

### 3.3. Transcript Purification. Time for Completion: Small Scale 4 h; Large Scale 5 h

1. *DNase digestion to remove the template DNA* (Figure 3A).
  - **small scale**: Continue with RQ1 DNase digestion by adding 10 mM Dithiothreitol, 20 U RNaseOUT, 5 U RQ1 DNase, 10 × RQ1 DNase buffer to a final concentration of 1×, supplement to 100 µL with RNase-free water, and incubate 30 min at 37 °C.
  - **large scale:** Continue with RQ1 DNase digestion by adding 10 mM Dithiothreitol, 100 U RNaseOUT, 25 U RQ1 DNase, 10 × RQ1 DNase buffer to a final concentration of 1×, supplement to 500 µL with RNase-free water, and incubate 1 h at 37 °C.
2. *Phenol/Chloroform/Isoamylalcohol extraction to remove the enzymes*.
  - **small scale**: For extraction, use RNase-free water to increase the reaction volume to 400–800 µL. Mix the sample with 1 volume Phenol/Chloroform/Isoamylalcohol (25:24:1), vortex, transfer into a Phase Lock Gel tube, and centrifuge 5 min, 20,000× g at room temperature. Transfer the aqueous upper phase into a new tube.
  - **large scale**: Distribute RQ1-digested transcripts to two 2 mL tubes. For extraction, use RNase-free water to increase the reaction volume to 800 µL each. Mix each sample with 1 volume Phenol/Chloroform/Isoamylalcohol, vortex, transfer into Phase Lock Gel tubes, and centrifuge 5 min, 20,000× g at room temperature. Transfer each of the aqueous upper phases into a new tube.
3. *Ethanol or isopropanol precipitation to remove residual chloroform and salts*. 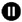
**PAUSE STEP:** Precipitate samples with 0.1 volumes 3 M NaAc (pH 6.5) and 2.5 volumes 100% ethanol or 0.7 volumes isopropanol at −20 °C for at least 30 min to overnight. Centrifuge at 4 °C for at least 20 min at 20,000× g. Wash the pellet with 70% ethanol and centrifuge again for 5 min, 20,000× g. Discard the supernatant completely, air dry the pellet briefly, and dissolve in 40 µL RNase-free water (**large scale**: 2 × 50 µL). **OPTIONAL STEP**: Mix 1% of the transcript with formamide gel-loading buffer and verify RNA integrity on denaturing polyacrylamide gel.
4. *Size exclusion chromatography to remove excess nucleoside triphosphates* (Figure 3A). Apply sample onto mini Quick Spin RNA Columns following the manufacturer’s instructions for gel filtration of the transcripts (**large scale**: use 2 columns per transcript); the sample volume increases to ~80 µL per column. Finally, measure the transcript concentration.

### 3.4. In Vitro Ciruclarization to Generate Circular RNA Sponges. Time for Completion: 17 h

1. *Annealing of transcript ends.* Within the annealing reaction, the double-stranded stem-loop (if included) is formed (or a splint oligonucleotide complementary to both ends of the transcript is annealed), thereby bringing 5′- and 3′-ends into close proximity, promoting an efficient circularization. This, together with an increased ligation reaction volume used in the next step, additionally favours intramolecular over intermolecular ligation reactions. **OPTIONAL STEP**: In this step, addition of equimolar amounts of a splint oligonucleotide can be useful if no stem-loop structure was included in the construct. Add 10× annealing buffer to a final concentration of 1× to purified transcripts and allow the annealing of the double-stranded stem-loop regions (or splint oligonucleotide) by incubating the samples for 2 min at 95 °C and decreasing the temperature by 1 °C/10 sec to 25 °C within a thermocycler.
2. In vitro circularization: ligation of 5′ and 3′-ends, thereby creating a covalently closed transcript.
  - **small scale**: Add 0.99 volume RNase-free water, 0.28 volume 10× reaction buffer for T4 RNA ligase (25 µL, 1× in final volume), and 40 U RNaseOUT (2 µL, 6.25 U/µL in final volume) to annealed transcripts. The volume should be 202.5 µL. Incubate for 10 min at 37 °C. Then, add 0.02 volume 10 mM ATP (5 µL, 0.2 mM in final volume), 0.15 volume DMSO (37.5 µL, 15% in final volume), and 50 U T4 RNA ligase (5 µL, 0.2 U/µL in final volume) for a total volume of 250 µL.
  - **large scale**: Scale up the above reaction two-fold to a total volume of 500 µL.
  Incubate at 16 °C overnight. Considering the activity of the T4 RNA ligase, there are three possible scenarios: (i) the transcript is not ligated and remains a linear monomer, (ii) the transcripts are ligated in an intermolecular way, resulting in the formation of dimerized (or multimerized) linear transcripts or (iii) the transcript is ligated in an intramolecular way, forming a covalently closed circular RNA molecule (Figure 3A,B). Increased reaction volumes with small concentrations of nucleic acids to be circularized [14]—amongst other techniques given in this protocol—contribute to preferentially intramolecular ligation events. In this fashion, circularization efficiencies reach between 40% and 60%.

### 3.5. Purification of Circular RNA Sponges. Time for Completion: 12 h

**Figure 4:**
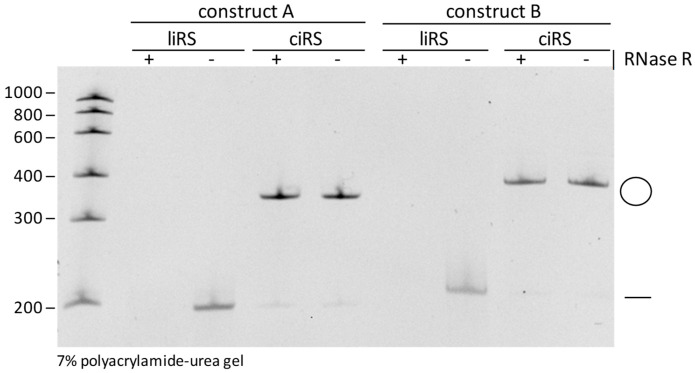
Validation of circularity by RNase R treatment. Both purified circular and linear RNA sponges (ciRS and liRS) of the two produced exemplary constructs with a length of 197 nt (construct **A**) and 208 nt (construct **B**) were subjected to RNase R exonuclease (+) or control (-) treatment and then analysed on 7% polyacrylamide-urea gel by ethidium bromide staining.

1. **OPTIONAL STEP**: In small scale circRNA production procedures, the following gel purification may be omitted and a preparative RNase R exonuclease treatment may be performed to digest remaining linear molecules and obtain exclusively circular RNA (directly to step 3.4.6). Note that rare larger multimeric circular RNA ligation products and RNase R-resistant linear RNA molecules remain if gel purification is omitted.
2. *Phenol/Chloroform/Isoamylalcohol extraction and ethanol or isopropanol precipitation are performed to ensure the purity of the reaction as previously described in Section 3.2 point 2 and 3.* The RNA pellet is dissolved in 50 µL formamide gel-loading buffer (**large scale**: RNA pellet is dissolved in 2 × 100 µL formamide gel-loading buffer).
3. *Polyacrylamide-urea gel electrophoresis for quality control and circRNA purification.* While the mobility of linear RNAs within polyacrylamide-urea gels is proportional to an RNA standard, circular RNAs show an aberrant mobility. Due to their circular conformation, the migration of the latter within polyacrylamide-urea gels is much slower compared to their linear counterparts. This aberrant migration can be compared between polyacrylamide gels of different acrylamide content and allows distinguishing linear di- or multimers from circular RNA—detectable as a size shift. Generally, the polyacrylamide content of the gels is dependent on the circRNA size. Referring to a circRNA size of ~200 nt, we recommend using 6% (300 V, 150 mA, 42 min) and 7% (300 V, 150 mA, 49 min) denaturing polyacrylamide gels, whereas circRNAs with a size of ~350 nt can be identified within 5% (300 V, 150 mA, 35 min) and 6% denaturing polyacrylamide gels. The optimal acrylamide concentrations should be determined experimentally for different RNA lengths. In order to analyse the circularization efficiency, 1% of the RNA transcript is loaded onto analytic denaturing polyacrylamide urea gels, followed by ethidium bromide (Figure 3B) or SYBR gold staining. The remaining 99% of the RNA mixture is loaded onto preparative denaturing polyacrylamide containing gel.
4. *RNA gel purification.* Visualization of RNA on a preparative gel is achieved by UV-shadowing upon transferring the gel onto a plastic-foil-wrapped thin-layer chromatography (TLC) plate coated with cellulose using a 254 nm fluorescent indicator. Irradiation with UV light at 254 nm excites the plate coating and results in a green fluorescence. The RNA on the contrary absorbs the UV light at this wavelength, casting a shadow against the fluorescent background. Note that the lower detection limit of this method is around 500 ng. Cut the circular RNA band (identified by the differential migration of circular RNA products in analytic gels of different acrylamide concentration) from the gel using a clean razor blade. 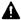 **CRITICAL STEP**—In order to increase the RNA purification efficiency, the excised polyacrylamide-urea gel fragments are to be crushed, as described in the following:
  - **Small scale**: Take a 500 µL safe-lock microcentrifuge tube. The latter is pierced in the centre of the bottom. A thin (e.g., 26 G) syringe needle is heated on a Bunsen burner flame to penetrate the plastic easily. In order to avoid injuries, we recommend extreme caution and suggest externally fixing the microcentrifuge tube in a tube stand within the course of piercing the tube, so no hands can be injured in the piercing process. Transfer the excised polyacrylamide-urea gel fragment into the pierced 500 µL safe-lock microcentrifuge tube and insert the latter into a fresh 1.5 mL Eppendorf tube. Centrifuge for 1 min at 20,000× g to crush the gel. 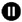 **PAUSE STEP:** Gel fragments can be stored at −80 °C overnight. Add 400–800 µL 1× PK-buffer to the crushed gel and incubate on a rotating wheel either overnight at room temperature or at 50 °C for 1 h. Freezing (e.g., using dry ice or liquid nitrogen) and thawing may increase yields. Use Costar Spin-X centrifuge tube filters to remove gel pieces by centrifuging for 1 min at 20,000× g.
  - **large scale**: Take a 15 mL Falcon tube, which is cleaved at the 3 mL sign using either strong scissors or a heated blade. In order to avoid injuries, we again recommend extreme caution and suggest externally fixing the tube within the course of cutting the tube, so no hands can be injured in the slicing process. The upper part of the tube is discarded, whereas the bottom is subjected to the piercing procedure as described above for the 500 µL tube. Transfer the excised polyacrylamide-urea gel fragment into the pierced lower 15 mL Falcon tube part and insert the latter into a fresh 5 mL Eppendorf tube. Centrifuge for 1 min at 20,000× g to crush the polyacrylamide-urea gel. 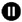 **PAUSE STEP:** Gel fragments can be stored at −80 °C overnight. Add 8 mL 1× PK-buffer to crushed gel, transfer this to the 15 mL Falcon tube, and rotate on a wheel either overnight at room temperature or at 50 °C for 1.5 h. Freezing with dry ice or liquid nitrogen and thawing in between may increase yields. Spin for 10 min at 20,000× g. Transfer the liquid into a fresh 15 mL tube, thereby preventing gel particle transfer.
5. *Phenol/Chloroform/Isoamylalcohol extraction and ethanol or isopropanol precipitation to purify and concentrate the produced circular RNAs.*
  - **small scale**: Follow the protocol described in Section 3.2 point 2 and 3. Circular RNA pellet—regularly yielding up 20 µg of *in vitro*-produced artificial circular RNA—is dissolved in an appropriate amount of RNase-free water, typically 20 µL.
  - **large scale**: Precipitate 8 mL supernatant with 0.1 volume 3 M NaAc (pH 6.5) and 0.7 volume isopropanol overnight at −20 °C. During the RNA precipitation procedure, residual SDS from the PK-buffer containing supernatant may not be soluble at very low temperatures. Incubate the solution on a wheel at 4 °C for 30 min. Afterwards, spin for 30 min at 20,000× g. Wash the pellet with 5 mL of 70% ethanol, spin for 5 min at 20,000× g, and take up RNA in 400 µL RNase-free water. Use Costar Spin-X centrifuge tube filters to remove remaining gel fragments by centrifuging for 1 min at 20,000× g. Resulting artificial circular RNA sponges regularly yield up to 350 µg, when preparation is conducted according to the large-scale protocol.
  Measure the ciRS concentration. Assess the final circRNA quality, as mentioned previously, by loading 1% of the preparation onto denaturing polyacrylamide gels of various polyacrylamide contents, followed by ethidium bromide or SYBR Gold staining (Figure 3C). 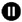 **PAUSE STEP:** CiRS can be stored at −80 °C. 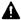 **CRITICAL STEP:** Autohydrolysis of RNA is a stochastically occurring event causing a cleavage of RNA molecules at a random position. Subjection of linear RNA to autohydrolysis results in a fragmentation of the RNA molecule. The latter is detectable within analytic polyacrylamide-urea gels as a typical smear after ethidium bromide or SYBR Gold staining. CircRNAs are characterized by an elevated stability compared to their linear counterparts [19]. Nevertheless, circular RNA molecules are degraded, but in contrast to their linear counterparts, in a biphasic manner. Thus, stochastic autohydrolysis cleaves the circRNA into a re-linearized isoform. Next, the re-linearized circRNA is in turn subjected to decay. Due to this fact, products of autohydrolytic degradation of efficiently purified circRNA sponges are detectable on analytic polyacrylamide-urea gels after ethidium bromide or SYBR Gold staining as faint bands of re-linearized RNA molecules within a circRNA preparation [16]. Therefore, excessive freezing and thawing of circRNAs should be avoided, as these may promote re-linearization by stochastic autohydolysis.
6. *Validation of circularity by Ribonuclease R (RNase R) treatment.* Despite the differential mobilities of circular RNAs within polyacrylamide-urea gels of different acrylamide contents, circularity of the produced sponges should also be proven by RNase R treatment. Incubate ciRS with 1 U/µg RNase R, 10 × RNase R Rxn Buffer to a final concentration of 1× and 40 U RNaseOUT, add to 40 µL with RNase-free water, and incubate for 20 min at 37 °C. Phenol/Chloroform/Isoamylalcohol extraction and ethanol or isopropanol precipitation are performed to purify the RNase R-treated circular RNAs. Assess circularity by loading 100 ng of the RNA onto a polyacrylamide-urea gel (Figure 4).
7. *Transfection of circRNA sponges in cell culture.***OPTIONAL STEP**: CiRS can be transfected into any eukaryotic cell culture-based system. Typically, transfection is performed using lipid- or synthetic-nanoparticles such as Lipofectamine 2000 or polyethylenimine formulations as used for siRNA transfections following the manufacturer’s instructions. Additionally, electroporation of circRNA sponges is possible. This procedure should use established protocols for the model system used.
